# The Impact of Different Visual Feedbacks in User Training on Motor Imagery Control in BCI

**DOI:** 10.1007/s10484-017-9383-z

**Published:** 2017-10-26

**Authors:** Dariusz Zapała, Piotr Francuz, Ewelina Zapała, Natalia Kopiś, Piotr Wierzgała, Paweł Augustynowicz, Andrzej Majkowski, Marcin Kołodziej

**Affiliations:** 1 Department of Experimental Psychology, The John Paul II Catholic University of Lublin, Lublin, Poland; 2 Institute of Computer Science, Maria Curie-Sklodowska University, Lublin, Poland; 3 Institute of Economics and Finance, Maria Curie-Skłodowska University, Lublin, Poland; 4 Institute of Theory of Electrical Engineering, Measurement and Information Systems, Warsaw University of Technology, Warsaw, Poland

**Keywords:** Brain–computer interface, Neurofeedback training, Sensorimotor rhythms, EEG, Motivation, Attention

## Abstract

The challenges of research into brain–computer interfaces (BCI) include significant individual differences in learning pace and in the effective operation of BCI devices. The use of neurofeedback training is a popular method of improving the effectiveness BCI operation. The purpose of the present study was to determine to what extent it is possible to improve the effectiveness of operation of sensorimotor rhythm-based brain–computer interfaces (SMR-BCI) by supplementing user training with elements modifying the characteristics of visual feedback. Four experimental groups had training designed to reinforce BCI control by: visual feedback in the form of dummy faces expressing emotions (Group 1); flashing the principal elements of visual feedback (Group 2) and giving both visual feedbacks in one condition (Group 3). The fourth group participated in training with no modifications (Group 4). Training consisted of a series of trials where the subjects directed a ball into a basket located to the right or left side of the screen. In Group 1 a schematic image a face, placed on the controlled object, showed various emotions, depending on the accuracy of control. In Group 2, the cue and targets were flashed with different frequency (4 Hz) than the remaining elements visible on the monitor. Both modifications were also used simultaneously in Group 3. SMR activity during the task was recorded before and after the training. In Group 3 there was a significant improvement in SMR control, compared to subjects in Group 2 and 4 (control). Differences between subjects in Groups 1, 2 and 4 (control) were insignificant. This means that relatively small changes in the training procedure may significantly impact the effectiveness of BCI control. Analysis of behavioural data acquired from all participants at training showed greater effectiveness in directing the object towards the right side of the screen. Subjects with the greatest improvement in SMR control showed a significantly lower difference in the accuracy of rightward and leftward movement than others.

## Introduction

Brain–computer interface devices make it possible to send information out of the body directly from the central nervous system (CNS), omitting endocrine and neuromuscular pathways. BCI systems are designed to register CNS activity in order to activate other devices or applications, which replace, restore, augment or supplement natural mechanisms of communicating with the environment. The entire process is based on the phenomenon of biological feedback (biofeedback), i.e. interaction between the user’s CNS and the external, as well as internal environment (Wolpaw and Wolpaw 2012). Brain–computer interfaces employ various techniques for recording brain activity, e.g.: fMRI (Posse et al. 2003; Yoo et al. 2004; Lee et al. 2009; Sitaram et al. 2011, 2012); fNIRS (Coyle et al. 2004; Naito et al. 2007; Sitaram et al. 2007); microelectrodes (Hochberg et al. 2006, 2012); EcoG (Leuthardt et al. 2004; Wilson et al. 2006; Schalk et al. 2008); and MEG (Mellinger et al. 2007; Buch et al. 2008; Broetz et al. 2010). However, some of these methods are less frequently used as a basis for developing BCI systems. Due to the low operational costs and satisfying ratio of temporal to spatial resolution, electroencephalographs (EEG) are the basis of approximately 60% of BCI systems tested in recent years (Hwang et al. 2013).

The operation of EEG-BCI is based on Steady State Visually Evoked Potentials (SSVEP) (Müller-Putz and Pfurtscheller 2008), P300 evoked potentials (Sellers et al. 2012) and sensorimotor rhythms (SMR)-BCI systems today predominantly employ the latter (Hwang et al. 2013) and such systems are known as SMR-BCI (Pfurtscheller and McFarland 2012). Sensorimotor rhythms are activations recorded on electrodes over areas of the sensorimotor cortex of the brain (Pfurtscheller and Lopes da Silva 1999). They are recorded in three frequency ranges: *μ* (8–12 Hz), *β* (18–30 Hz) and *γ* (30–200 + Hz), although the detailed limits of frequency ranges tend to be defined individually (Pineda 2005).

The phenomenon of SMR oscillations takes two forms: a decrease in SMR power during movement, that is the so-called event-related desynchronization (ERD) (Pfurtscheller and Aranibar 1979) and an increase in power after completing the movement, i.e. event-related synchronization (ERS) (Pfurtscheller 1992). The phenomenon of ERD/ERS occurs during the actual movement of a single finger, the whole hand, foot, and tongue as well as while observing or imagining such movement (Pineda 2005; Pfurtscheller et al. 2006). In the case of hand movements (whether imagined or actual) the effects of ERD/ERS occur more strongly on C3 or C4 electrodes located contralaterally to the hand involved in the task (Pfurtscheller and Lopes da Silva 1999).

SMR-BCIs are employed to control such devices as wheelchairs (Huang et al. 2012), prosthetic hands (Pfurtscheller et al. 2000), and robot arms (Ang et al. 2010), as well as software, which enables the writing of messages (Müller and Blankertz 2006), control of a cursor on the computer screen (Wolpaw et al. 1991) or a virtual avatar (Leeb et al. 2007).

The effectiveness of SMR-BCI control depends on individual factors and subjects participating in the same training do not all achieve identical results (Curran and Stokes 2003). Indeed, the reported phenomenon of BCI illiteracy, otherwise known as BCI aphasia (Kübler and Muller 2007), indicates a subject’s inability to control a given type of device at a level exceeding random success, despite training. The problem affects approx. 15–30% of those participating in research utilising brain–computer interfaces. Research currently being conducted into psychological (Blankertz et al. 2010; Hammer et al. 2012) and neurophysiological factors (Grosse-Wentrup and Schölkopf 2012), aims to improve the effectiveness of SMR-BCI control through the use of suitable training programmes (Hwang et al. 2009; Zapała et al. 2015).

Neurofeedback training an essential element of learning to operate SMR-BCIs, provides a simplified model of a situation in which the user controls a BCI (Neuper and Pfurtscheller 2010). The purpose of neurofeedback training is to learn to control one’s own mental activity so that, based on observation of the response elicited by such activity, one can achieve the best possible control of the interface. In order to optimise the effects of training, modifications are introduced in the types of tasks (McFarland et al. 2010; Krausz et al. 2003), instructions (Bonnet et al. 2013), method of providing feedback (Hwang et al. 2009; Kaufmann et al. 2011) and/or duration of specific training sessions and trials (McFarland et al. 2010). The parameters of these procedures, however, are defined in an arbitrary manner and most frequently disregard other processes involved in the mechanism of learning the skill of controlling one’s own brain waves (Lotte et al. 2013).

Studies focusing on variables modifying SMR-BCI control constitute a small percentage of the research concerning the enhancement in performance of such interfaces (Hwang et al. 2013). Nevertheless, it has been established that psychological variables significantly impact the effectiveness of SMR-BCI control (Nijboer et al. 2010; Hammer et al. 2012; Jeunet et al. 2015). Such factors can also be modified during neurofeedback training (Lotte et al. 2013; Barbero and Grosse-Wentrup 2010; Koerner et al. 2014). Researchers believe that by reinforcing selected cognitive and motivational functions in SMR-BCI users, so that they continue to learn to control such interfaces, it may be possible to significantly improve effective operation of such devices (Leeb et al. 2007; Lotte et al. 2013). Currently, studies into factors impacting effectiveness of the SMR-BCI operation mostly employ standard training procedures, such as the *Graz-BCI* protocol, and their modifications (Jeunet et al. 2015). Therefore, it seems worthwhile to examine in what way new elements introduced into neurofeedback training would impact SMR-BCI performance.

The present study has been designed to answer the question whether it is possible to achieve more effective SMR-BCI control by modifying visual feedback in user training. We put forward the hypothesis that by supplementing the training procedure with features which made it easy for the subjects to focus their attention on the essential elements of each trial, such as the visual cues and goal of the task, it would be possible to increase control over the skill being practised. The subjects’ focus can be increased for instance by making sure that the field of vision contains objects with a so-called priority feature (McLoad et al. 1991) which differentiates the objects from other elements on the screen. If the subject is asked to direct a moving object (e.g. a ball) to a target (e.g. a basket) placed on the board, the key elements (such as the ball and the basket) should be highlighted, for example with blinking at a specified frequency, to distinguish them from the other objects which are not important for the proper performance of the task.

Accuracy during a task may be reinforced by providing feedback about the subject’s current performance (Krausz et al. 2003). It is important to make sure that the relevant information be given in a way which is emotionally engaging for the subject (Curran and Stokes 2003). In our study we were reinforcing subjects’ motivation to continue training by providing them with additional information about their performance in each trial in the form of schematic images of faces expressing sadness or joy. Application of emotional feedback is justified by some earlier studies which reported that users’ positive emotional state was a factor increasing motivation for BCI control (Nijboer et al. 2008). Furthermore, schematic images of faces have already been employed to improve effectiveness in the control of P300-BCI (Chen et al. 2015).

We also expected that the effectiveness of the training would be more strongly influenced if the training procedure incorporated elements affecting both the attention and motivation of the subjects in comparison to a situation when only one of these factors was applied. This assumption was inspired by a study published by Leeb and colleagues (2007) suggesting that subjects’ willingness to continue BCI control training increases if the virtual environment is visually more complex. By combining the feedback presented in the form of faces expressing emotions with objects highlighted to stand out from the background it is possible to create an environment which is visually more complex than if these two features are applied separately. We assumed that in comparison to the other groups accuracy of control would be higher in the study group in which both modifications were used simultaneously.

Lack of a modification of the feedback method in the control group was expected to result in the smallest increase in the control of sensorimotor rhythms. The basic training scheme is similar to the standard *Graz-BCI* (Neuper and Pfurtscheller 2010) and *Wadsworth-BCI* procedures (Wolpaw et al. 1991).

The hypotheses were verified in the course of a multi-stage empirical study comprising neurofeedback training with a BCI set, and the measurement of sensorimotor rhythms during stationary EEG registration. The effectiveness of sensorimotor rhythm control was operationalised with the use of two indicators: behavioural, i.e. number of responses complying with the instruction (online mode), and physiological. A physiological indicator of the effectiveness of SMR-BCI control is acquired by comparing changes in μ (8–12 Hz) and β (13–30) sensorimotor rhythms, before and after training (offline mode).

Both bands of sensorimotor rhythms can be modulated by various processes accompanying motor activity. The lower band is generally linked with processes involved in simple movements, and with the mental simulation of movements of hand, foot or tongue. (Pfurtscheller et al. 2006). Oscillations in the range of β waves are linked with imagining complex movements and those requiring engagement (Nakagawa et al. 2011), and the control of various parameters of movement (Zaepffel et al. 2013). We expected that separate analysis of both frequency bands would show differences in β waves, because neurofeedback training should lead to increased engagement and improved accuracy of the mentally simulated movement.

According to some researchers skill in SMR control may be acquired gradually, e.g. from learning to create images of one hand, to reaching the ability to create images of both hands (Wolpaw and McFarland 2004; Pfurtscheller et al. 2006; McFarland et al. 2010). Because of this, in our study SMR desynchronization at offline and online stage was analysed separately for imagined movements of the right and left hand.

Differences in the strength and topography of the ERD/ERS effect during imagery of right and left hand movement were demonstrated for instance, in a study by McFarland and colleagues (2000), yet the effects of such differences in the accuracy of SMR-BCI control have rarely been examined. The present study was designed to investigate whether there were statistically significant differences in the accuracy of the actual leftward and rightward control of the object between participants who were found to have the highest and the lowest increase in SMR control skills following the training. We expect that individuals failing to gain improvement in SMR control as a result of the training, who potentially are BCI-illiterate, also face difficulties in creating different motor imagery. Individuals presenting with poorer SMR-BCI performance are also less capable of creating vivid kinesthetic imagery (Vuckovic and Osuagwu 2013; Marchesotti et al. 2016). Those affected by BCI illiteracy face difficulties creating separate imagery for both hands which results in one-sided control; they may also be found with indistinct changes in SMR, which results in poorer BCI control.

In accordance with the “gold standard” proposed by McFarland and Krusienski (2012) for assessing the effectiveness of testing a new BCI method, the most valuable results are obtained if both online and offline measures are applied in one test. The findings acquired in bouth offline and online modes were subjected to the same signal processing procedure in order to examine the compatibility of the results.

Earlier studies suggested a significant relationship between vividness of motor imagery and SMR-BCI performance (Vuckovic and Osuagwu 2013; Marchesotti et al. 2016), and because of this the level of this variable was examined during recruitment for the study and at the stage of assigning the subjects to experimental groups. The analyses took into account the subjects’ gender as one of the inter-subject factors.

## Methods

### Participants

The experiment was carried out with 40 subjects (20 women) aged from 20 to 27 (*M* = 22.60; *SD* = 1.72). One of the participants reported being left-handed. All the subjects were volunteers who gave their written consent to take part in the study and declared they were not taking medication or other psychoactive substances on a permanent basis. Motor imagery ability was controlled with the Movement Imagery Questionnaire-Revised Second Version (MIQ-RS) (Gregg et al. 2010) and was calculated separately for: visual motor imagery—VMI (*M* = 6.04; *SD* = 0.64) and kinesthetic motor imagery—KMI (*M* = 5.77; *SD* = 0.87). Experimental groups were not significantly different in terms of: VMI [*F*(3,36) = 0.47, *p* = 0.703] or KMI [*F*(3,36) = 1.26, *p* = 0.303]. At the end of the whole procedure, the participants were each paid a remuneration of 20 USD. The Ethics Committee of the Institute of Psychology approved of the study.

### Apparatus

Measurement of changes in the activity level of sensorimotor rhythms were carried out with EEG system GES 300 (Electrical Geodesics, Inc. Eugene, OR, USA), comprising Net Amps 300 amplifier (output resistance 200 MΩ; recording range from 0.01 to 1000 Hz). The recording was made with the use of a 128-channel cap with passive electrodes HydroCel Geodesic Sensor Net (resistance during recording < 30 kΩ). Data sampling during examination was defined at 500 Hz, and the recording was made with the use of Net Station 4.4 (EGI, Eugene, OR, USA). The experimental procedure was designed and displayed on the screen with the use of E-Prime, version 2.0 (Psychology Software Tools, Pittsburgh, PA, USA).

Neurofeedback training was carried out using the Discovery 24E DC amplifier, from BrainMaster Technologies, Inc. (Bedford, OH, USA) with output resistance below 1000GΩ and recording range from 0.000 to 1000 Hz. The recording was carried out with the use of 10-cup passive gel electrodes, Ag/AgCl (C3; C4; FC3; FC4; C5; C1; C2; C6; CP3; CP4) with a right-ear reference electrode and ground electrode placed on a left ear. Recording and processing of the data in online mode was carried out in OpenViBE 0.18.0 (Inria Hybrid Team, Rennes Cedex, France) with data sampling 125 Hz. The signal was filtered with bandpass filter (8–30 Hz). The data were also subjected to spatial filter Common Spatial Pattern (CSP). Signal classification was carried out with the use of Linear Discriminant Analysis (LDA) (Lugger et al. 1998). The training programmes were based on application systems prepared in C++ programming language.

During EEG measurement, the stimuli were displayed on an LCD screen, with a diagonal measurement of 17 inches, resolution of 1280 × 1024 pixels, and during the neurofeedback training on a 23 inch screen, resolution of 1920 × 1080 pixels. The subjects were seated at a distance of 60 cm from the monitor. Introductory processing of EEG records was performed with EEGLab v12.0.2.6b, upgraded to MATLAB 7.9.0 (MathWorks, Natick, MA, USA), and the statistical analysis as well as visualization of the results with STATISTICA 12 (StatSoft, Inc., USA).

### Procedure

The experimental stage comprised an initial measurement (“Before”) which involved the recording of sensorimotor rhythm activity during an imagined hand movement. This was followed by a change in the device and training procedure (neurofeedback). The same pattern of recording was repeated after the training session as the final measurement (“After”).

#### EEG Recording

**Fig. 1:**
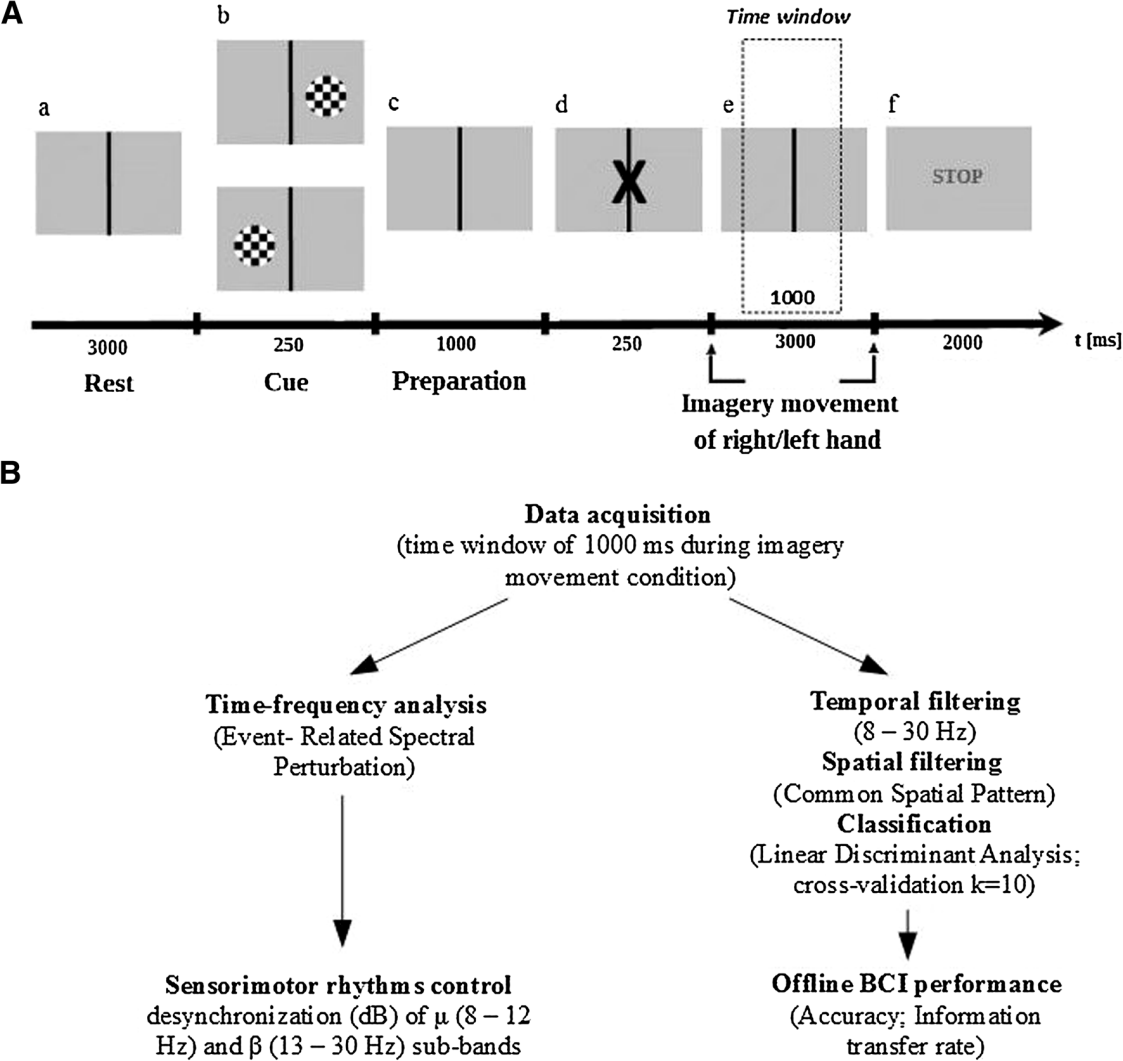
**A** Experimental procedure. *a* The subject is not performing any activity; *b* a cue is displayed and its location shows what movement should be imagined by the subject after the task starts; *c* time for preparing to begin the task; the subject is not performing any activity; *d* X sign starts the imagery task; *e* the subject imagines the movement of clenching his/her right or left hand, in accordance with the cue; *f* end of the imagery task. **B** Signal processing scheme (offline mode)

The procedure for recording the EEG during the performance of a mental imagery task was developed by modifying the paradigm proposed by Hwang, Kwon and Im (2009). The data were registered from the moment a visual cue appeared until the end of the performance of the imagery task (Fig. 1). During the recording undertaken before and after neurofeedback training, each subject performed a total of 180 trials (90 trials each for imagined left and right-hand movement). The trials were displayed for each participant at random.

#### Neurofeedback Training

**Fig. 2:**
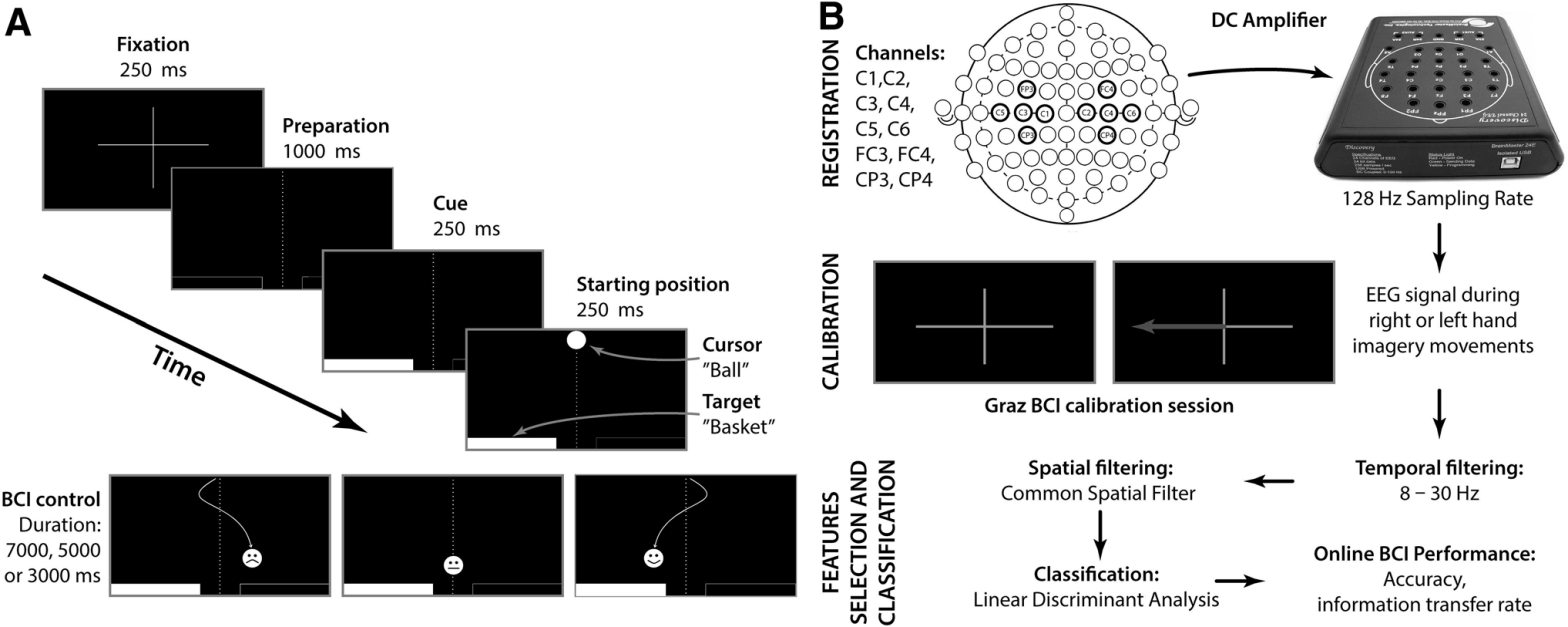
**a** Neurofeedback training pattern. The control stage presented the possible responses of the users (response matching the cue, no response, non-complying response) and the elements modified during the study (ball and basket). **b** Signal processing scheme (online mode)

The training pattern was a modified version of the procedure used by Krausz et al. (2003) which is presented in Fig. 2a. The training of each participant consisted of trials lasting for a few seconds, during which the participant was asked to lead a ball, falling from the top to the bottom of the screen, to one of the marked baskets. After the ball was placed in one of the baskets, or fell freely away from the baskets, the next trial started. After 40 trials (constituting a series) were displayed, there was a short pause (its length depended on the subject). Each series was repeated 4 times, which gave a total of 160 trials, after which there was another pause (4 series and 4 pauses constituted one session). During the entire neurofeedback training the subject performed a total of 480 trials (240 for each hand).

The subjects were divided into four independent groups, corresponding to the type of modification introduced in the training pattern.

In Group 2 the ball and the highlighted basket were blinking with a frequency of 4 Hz, so that the elements stood out against the remaining objects displayed on the screen during training. In the variant in Group 1, the subject received ongoing feedback about the progress of his/her performance. This information was provided to the subject in the form of a face expressing various emotional states, as displayed on the control ball. At the start, the face exhibited a neutral expression. A ball which fell with no user control or was led in a wrong direction showed sadness. A ball that led towards a correct basket showed a smile (Fig. 2a).

In Group 3 both modifications were used simultaneously, i.e. the blinking of the ball with a frequency of 4 Hz and the additional feedback. Group 4 (control) participated in training without any additional features.

### Data Acquisition and Processing

Electrophysiological data (initial and final EEG measurements) were pre-processed, first with the use of a high-pass filter below 1 Hz, and a low-pass filter over 40 Hz (FIR filter); that was then followed by a filtering out of the recording corresponding to the frequency matching the operation of power lines—50 Hz (Notch filter). After filtering, the data were subjected to the process of re-referencing the signal (CAR, common average reference) for all channels except those closest to the eyes (electrodes: 1, 14, 17, 21, 25, 32, 125, 126, 127, 128). Ocular and muscular artefacts were removed using an independent component analysis (ICA) procedure. The blink-related components of ICA were identified for every subject individually, based on their specific time courses (i.e., brief, large monopolar potentials) and heuristic stating that the eye components project strongest at frontal sites. The lateral eye movement components of ICA were identified based on the assumption that such components should project strongest to far frontal sites, and should show a polarity difference between the two periocular sites (see: Jung et al. 2000a, b). Such components were removed from the data.

The data prepared in this way were divided into segments matching the period of time from the disappearance of the visual cue to the end of the imagery task. Calculations for the experimental conditions (event) were performed for the temporal window with a length of 1 s, counted for the performance of imagery task (Fig. 1) and they were subjected to time–frequency decomposition with Event-Related Spectral Perturbation (ERSP) (Makeig 1993) in order to calculate the signal strength (dB) for the entire window (Formula 1).

1 $$ERSP(f,t)=\frac{1}{n}\sum\limits_{{k=1}}^{\upmu } {{{\left| {{F_k} (f,t)} \right|}^2}}$$

Formula 1 shows the Event-Related Spectral Perturbation equation. For *n* trials, if *F*_k_(*f, t*) is the spectral estimate of trial *k* at frequency *f* and time *t* (Delorme and Makeig 2004). To compute *F*_k_(*f, t*) we used a sinusoidal wavelet transformations (3-cycles; 0.5 s).

The analyses took into account recordings from 12 channels covering the right and left motor cortex area in the HydroCel Geodesic Sensor Net cap (Bernier et al. 2007). Comparative analyses related to the task involving right-hand motor imagery were based on recordings from six electrodes located on the contralateral side (electrodes corresponding with C3 location: 36, 37, 41, 42, 53, 54). Likewise, for left-hand movement, the recording from channels on the opposite side were used (electrodes corresponding with C4 location: 79, 86, 87, 93, 103, 104). The procedure was applied to two frequency ranges: µ (8–12 Hz), β (13–30 Hz).

In additionally SMR desynchronization before and after neurofeedback training was averaged for the entire frequency band, separately for the rightward and leftward movement and for both tasks simultaneously. Based upon this, we identified two groups of subjects who achieved the greatest decrease (*N* = 10) and the greatest increase in SMR power (*N* = 10). We can say that the subjects in the former group improved with respect to SMR control while in the latter group, the function deteriorated in comparison to the initial measurement.

Electrophysiological data were also subjected to a separate offline analysis to enable a comparison of the session results (“Before” and “After”) with the training procedure results. The analyses were carried out for ten electrodes (FC3, C1, C3, C5, CP3, CP4, C2, C4, C6, FC4). EEG signals were filtered using a FIR filter with passband 8–30 Hz. In the next step, the EEG fragments associated with imagining the right and the left hand movement were separated forming EEG data for two classes. For signals associated with imagining the right and the left hand movement, a Common Spatial Pattern (CSP) filter was applied (Ramoser et al. 2000). In this method, new signals are formed by spatial filtering, which enables the maximising of variances of signals representing the two different classes.

In our experiment each component, following application of the CPS filter, was divided into 1-second windows overlapping in 0.5 s. The logarithm of power was calculated for each window. In this way, we received a feature vector that was used for signal classification (2 classes—imagining the right and the left hand movement).

Classification was performed using an LDA classifier (Lugger et al. 1998). The data were divided into learning and testing sets with a10-fold cross validation test. At the end the information transfer rate (ITR) was calculated for each user session (Formula 2).

2 $$ITR=\frac{{60}}{{{T_{act}}}}\left( {lo{g_2}N+{p_a}lo{g_2}{p_a}+\left( {1 - {p_a}} \right)lo{g_2}\frac{{1 - {p_a}}}{{N - 1}}} \right)$$

Formula (2) shows the information transfer rate (ITR) equation. ITR for a BCI system with *N* possible choices (e.g. mental tasks), with an average efficacy expressed as *p*_*a*_ and time to make a choice as *T*_*act*_ (in seconds) (Shannon and Weaver 1949). ITR (expressed in bits per minute) is defined as the amount of information transferred via a BCI in a time unit. It combines both the speed and effectiveness of the interface.

The signal during neurofeedback training was processed in real time, in the same way as the EEG data from measurement (“Before” and “After”) (Fig. 2b). Behavioural data were acquired via the automatic recording of correct responses during the neurofeedback training. Points were given if the ball hit any part of the highlighted basket area. No point was scored if there was no response (the ball fell down in the area between the baskets) or if the ball was placed in an area away from the highlighted basket. The results were recorded for accuracy (number of responses consistent with the instruction) and in the form of ITR.

## Results

### Offline Mode

The repeated measure analysis of variance with GROUP (1–4) and SEX (Female, Male) as inter-subject factors and two within-subject factors: the imagined HAND (left vs. right) and MEASUREMENT (before vs. after) was performed separately on two sub-bands within the range of sensorimotor rhythms: μ = 8–12 Hz and β = 13–30 Hz.

**Fig. 3:**
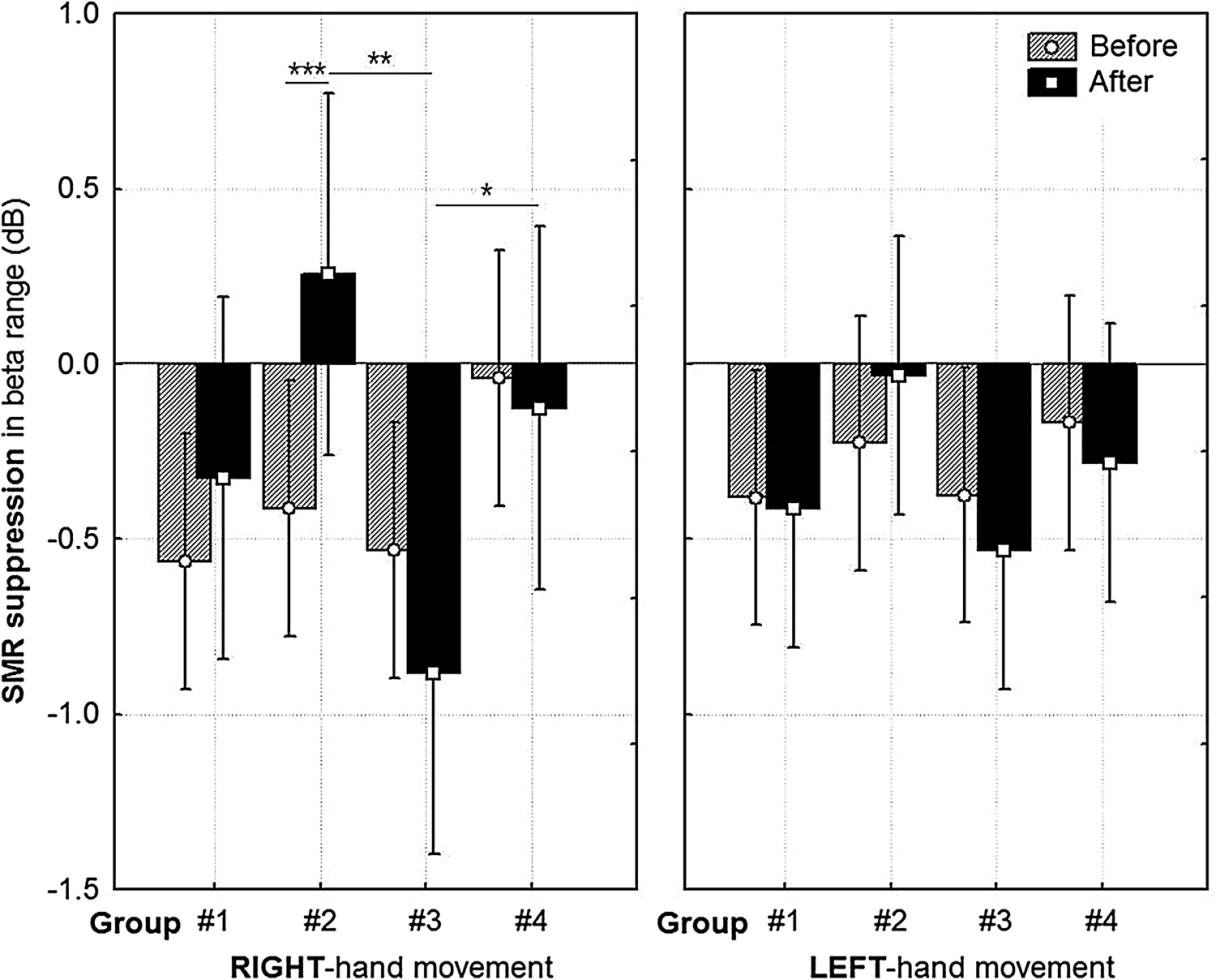
Differences in desynchronization of β bands (13–30 Hz), related to the effect of HAND × GROUP × MEASUREMENT, for the imagery hands movements. The vertical bars show 0.95 confidence intervals. Significant differences in *post hoc* comparisons with the Bonferroni correction are marked with brackets: *p = 0.04; **p = 0.003; ***p < 0.001

**Fig. 4:**
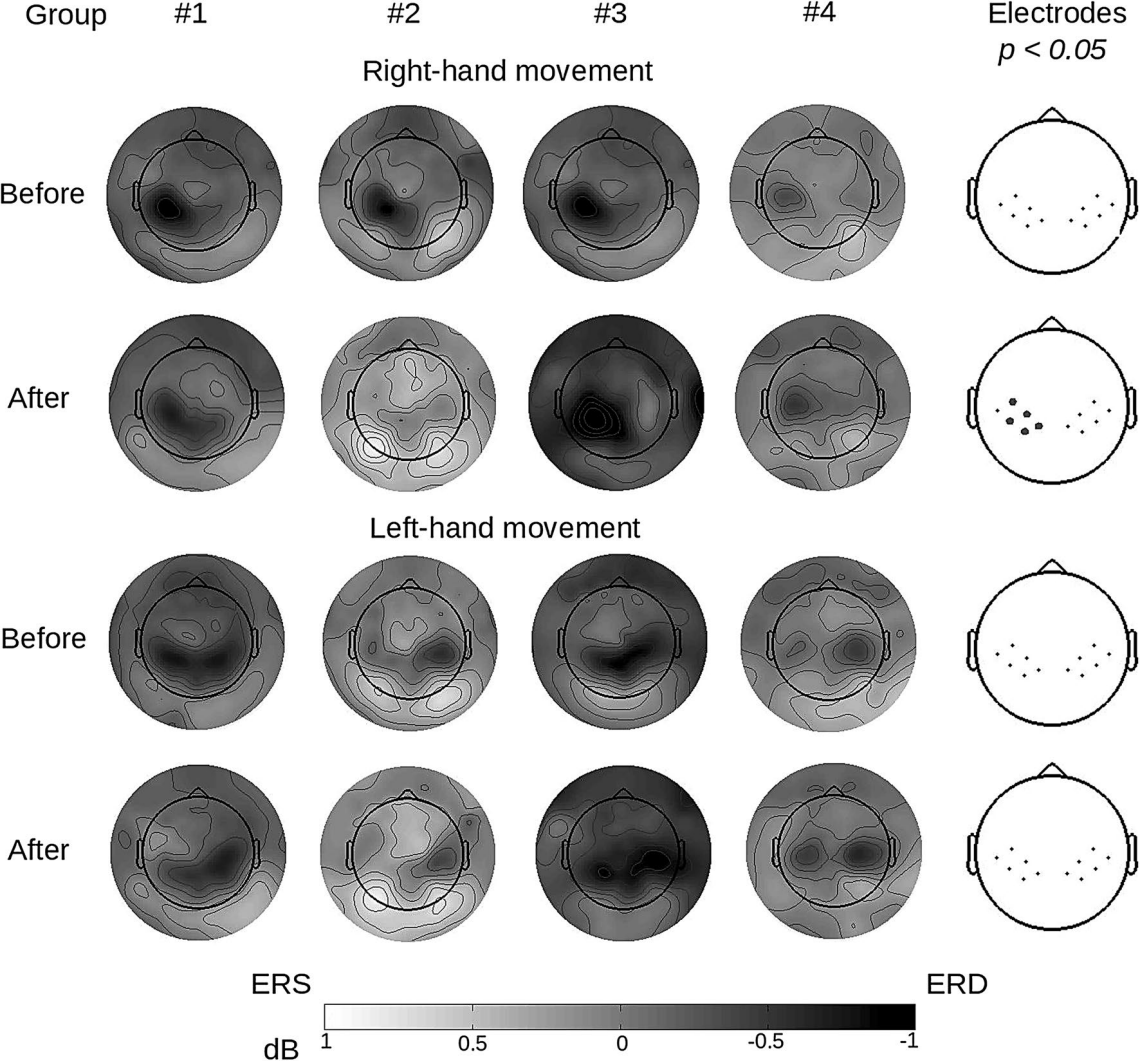
Maps of SMR distribution on the skull (8–30 Hz) during imagined movement of the right and left hand. Electrodes shown on the right registered statistically significant differences in the strength of signal between the experimental groups (marked with grey dots). There is a visible lack of differences before the training procedure was applied

There were no significant main effects or interaction for μ-frequency: HAND *F*(1,32) = 0.49, *p* = 0.488, *η*_*p*_^2^ = 0.01; GROUP *F*(3,32) = 1.22, *p* = 0.318, *η*_*p*_^2^ = 0.1 SEX *F*(1,32) = 0.43, *p* = 0.514, *η*_*p*_^2^ = 0.01; MEASUREMENT *F*(1,32) = 0.02, *p* = 0.901, *η*_*p*_^2^ = 0.004 and HAND × GROUP × MEASUREMENT × SEX *F*(3,32) = 1.18, p = 0.333, *η*_*p*_^2^ = 0.09. Also, there were no main effects or interaction in the β—range, HAND *F*(1,32) = 0.26, *p* = 0.616, *η*_*p*_^2^ = 0.007; GROUP *F*(3,32) = 1.75, *p* = 0.177, *η*_*p*_^2^ = 0.14 SEX *F*(1,32) = 0.02, *p* = 0.874, *η*_*p*_^2^ = 0.0008; MEASUREMENT *F*(1,32) = 0.25, *p* = 0.621, *η*_*p*_^2^ = 0.008 and HAND × GROUP × MEASUREMENT × SEX *F*(3,32) = 0.568, *p* = 0.639, *η*_*p*_^2^ = 0.05, but a significant effect was observed between the factors of HAND × GROUP × MEASUREMENT *F*(3,32) = 3.20, *p* = 0.035, *η*_*p*_^2^ = 0.23. The calculated *post hoc* comparisons with Bonferroni correction confirm that the Group 3 (dummy face + flashing elements) after the training stage (MEASUREMENT = after), presented stronger suppression than was the case with Group 2 (*p* = 0.003) and Group 4 (*p* = 0.04). Moreover, the results of Group 1 (*p* = 0.58) and Group 2 (*p* = 0.29) did not differ from the results of Group 4. In Group 2, the suppression during the second measurement was weaker than before NF session (*p* < 0.001) (Fig. 3). The effect was obtained for the task of the imagery right-hand movement. The distribution of signal power is shown in Fig. 4. Notably, *post hoc* comparisons only showed differences between the experimental groups after the training; *F*(3,32) = 3.48; *p* = 0.02, *η*_*p*_^2^ = 0.23 Before the user training stage there were no significant effects in β suppression during imagery left *F*(3,32) = 0.36; *p* = 0.77, *η*_*p*_^2^ = 0.03 or right, *F*(3,32) = 1.81; *p* = 0.16, *η*_*p*_^2^ = 0.13 hand movements. This supports the assumption that the differences between the groups are a result of training.

### Online Mode

The analysis of variance, with GROUP (1–4) and SEX (Female, Male) as inter-subject factors and a run on behavioural data (accuracy score) acquired during the stage of neurofeedback training did not show statistically significant main effects or interactions of GROUP: *F*(3,36) = 1.11, *p* = 0.36, *η*_*p*_^2^ = 0.09 ; SEX: *F*(1,39) = 0.22, *p* = 0.641, *η*_*p*_^2^ = 0.01 and GROUP × SEX: *F*(1,39) = 0.93, *p* = 0.438, *η*_*p*_^2^ = 0.08.

**Fig. 5:**
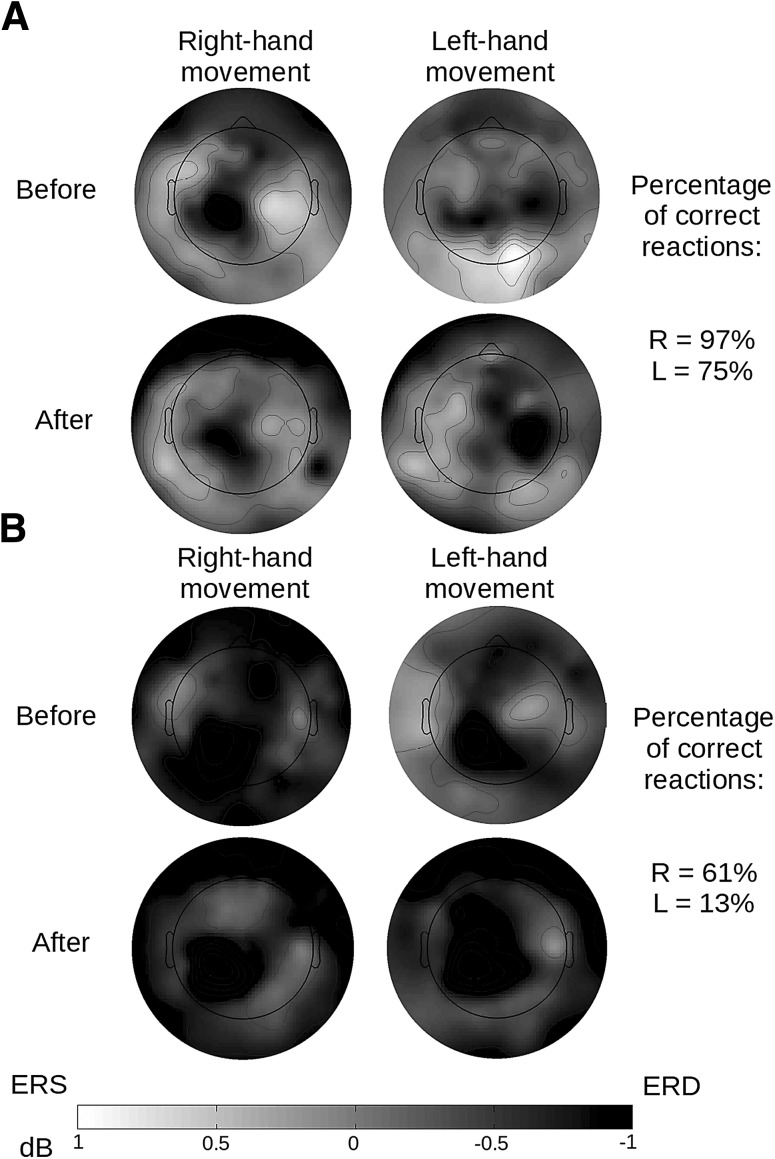
Sample maps showing the distribution of SMR on the skull (8–30 Hz) during imagined right and left-hand movement: **a** a subject with a small difference in accuracy of performing both training tasks during the first and second EEG measurement was found with desynchronization on the appropriate side, contralateral in relation to the cue displayed; **b** a subject who at the training stage tended to correctly hit the basket if the target was situated on the right side, in EEG measurement “Before” and “After” was found with desynchronization exclusively on the left side, regardless of the task

Analysis of the data related to control accuracy during training with the non-parametric Mann Whitney *U* test showed that the subjects with the most pronounced decrease in band power in comparison to the first EEG recording (*N* = 10; *M* = − 0.78 dB; *SD* = 0.215 dB) achieved lower disparity between the results in rightward and leftward control than the subjects who experienced an increase in wave strength at the same time (*N* = 10; *M* = 0.99 dB; *SD* = 0.386 dB) *U* M-W = 18; *p* = 0.015. The subjects found to have the strongest effects from the training achieved similar accuracy in both tasks (*Md* = 45.50), while those with the poorest results were more successful in one type of movement (*Md* = 132). Figure 5 presents the topography of the ERD/ERS effect relative to the size of difference in accuracy during the training session.

**Fig. 6:**
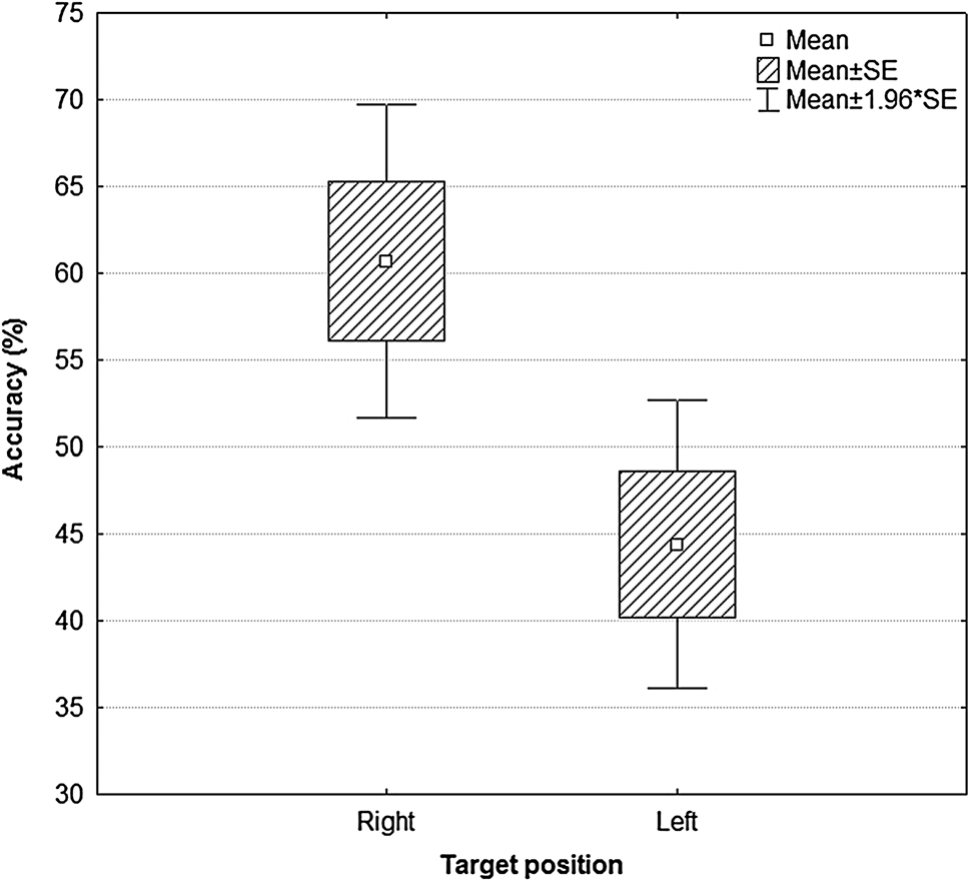
Percent of correct reactions presented by users per type of TASK

A *t* test revealed significant differences in successful performance dependent on the specific task during training *t*(1,39) = 2.06, *p* = 0.046. The subjects more often correctly hit the target situated on the right (*M* = 60.70; *SE* = 29.09) than on the left (*M* = 44.38; *SE* = 26.7). The effect was observed during all training sessions and in all the experimental groups (Fig. 6).

ITR from the training session shows a positive correlation with the difference in the accuracy of the imagery classification (“After”–“Before”) calculated from EEG measurement data subjected to the same procedure as the signal from the neurofeedback session *r* = 0.37, *p* = 0.02. This means that the subjects’ greater ability to control movement during sessions with feedback coincided with higher improvement in SMR control recorded during offline registration.

## Discussion

As expected, after neurofeedback training, Group 3 (dummy face + flashing elements) presented with greater suppression during imagined movement than Group 4, which participated in training with no modifications. Furthermore, Group 3 achieved significantly better results during the second measurement in comparison to Group 2 subjected to the training procedure with a single modification (flashing elements). The findings relating to the second group were the opposite of those expected. After the relevant training procedure was employed, there was a significant decrease in suppression of sensorimotor rhythms, an effect of its key importance in SMR-BCI control. This specific procedure may have resulted in a greater level of fatigue in the users than the remaining variants. Fatigue during the observation of objects displayed at low frequencies is a common problem in SSVEP (Cao et al. 2014) and P300 BCI (Fazel-Rezai 2007). In this context it seems interesting that in Group 3 the same flashing movement had no negative impact on the effects of training. Moreover, following the training procedure accompanied only with a display of a face showing emotions, Group 1 was found with suppression of SMR which did not differ significantly from that recorded in either the group training with no modifications (Group 4) or the group whose training comprised two new elements (Group 3). It is likely that when it was applied alone, the flashing movement caused fatigue as it attracted attention only to the objects which were moving on the screen yet carried no additional information for the user. A schematic image of a human face flashing at a low frequency is applied in P300-BCI to decrease fatigue and improve the mental condition in subjects using this kind of interfaces (Chen et al. 2015). Introducing the same elements into SMR-BCI training tested in the present study may have produced a similar, positive effect.

All the significant differences in offline analyses were related to the upper band of sensorimotor rhythms (β), which may be evidence of participants’ increased control over imagery movement. Decreased activity in β oscillations during motor tasks is observed in subjects with reduced motor control, e.g. in neurological disorders (Leocani and Comi 2006). The upper SMR band is also linked to more difficult tasks or those requiring greater engagement (Nakagawa et al. 2011). Changes in this range of frequencies may suggest that the training also led to improved accuracy with which the participants simulated the imagery movement.

The evidence obtained is limited to electrophysiological measurements and tasks involving right-hand motor imagery, yet it is consistent with the findings of other experiments (Leeb et al. 2007; Jin et al. 2012; Kober et al. 2013). Moreover, the imagined movement of the dominant hand is used in some experiments during the initial SMR-BCI sessions (McFarland et al. 2010) or in assessing the vividness of the imagery (Neuper et al. 2005). In the case of right-handed individuals, who accounted for > 97% of the participants, left-hand motor imagery may be a more challenging task, which may explain the lack of effects following a singiel training session.

Lateralisation of the task was also important for accuracy in online mode. It was observed that when the target was on the right side of the screen, the subjects hit it with the ball more frequently than if the left-side platform was displayed. The participants in the study who, regardless of the type of training, achieved the lowest improvement in SMR control between sessions, were also found with the greatest differences in rightward and leftward control accuracy in online mode. Although differences in the topography of sensorimotor waves between the imagined right-hand and left-hand movement have already been reported (Stancák and Pfurtscheller 1996; McFarland et al. 2000), the direct effects of topography in online BCI control require further investigation.

It should be emphasised that although the experiment was conducted in two stages (offline and online), there was significant correlation found beetween the results obtained that were subjected to the same signal processing procedure. This is consistent with earlier studies which suggest that measurement of sensorimotor waves during imagined movement permits the prediction of the effectiveness of BCI performance in real time (Blankertz et al. 2010).

## Conclusion

In summary, the present study has demonstrated that it is possible to influence SMR-BCI control by manipulating the visual elements used in the procedure of neurofeedback training. The findings related to the improved control of SMR desynchronization in Group 3 to justify investigating a possible level of complexity of the neurofeedback procedure, in terms of the applied visual elements, which would effectively improve an individual’s capacity to learn BCI control. Trials using complex graphical environments, such as three dimensional stimuli (Leeb et al. 2007; Scherer et al. 2008), or those acting on a several senses of the user at the same time (Sollfrank et al. 2016), justify the assumption that one training procedure may incorporate a number of elements which affect various mental processes involved in learning. Complex neurofeedback training based on multiple media, and designed to simultaneously reinforce a number of mechanisms involved in the process of motor imagery control, may prove to be another step towards overcoming the phenomenon of BCI illiteracy.

The observed difference in the accuracy of performance between tasks involving rightward and leftward movement based on real-time BCI experiments has not previously been reported. Therefore, there seem to be good reasons for further studies to investigate whether lateral dominance may have an impact on the effectiveness in using brain–computer interfaces based on imagery involving lateralised movement.
